# Application of Cameriere’s method for dental age estimation in children in South China

**DOI:** 10.1080/20961790.2020.1830515

**Published:** 2021-01-04

**Authors:** Zedeng Yang, Dan Wen, Jiao Xiao, Qianying Liu, Shule Sun, Aliye Kureshi, Yunfeng Chang, Lagabaiyila Zha

**Affiliations:** a Department of Forensic Science, School of Basic Medical Sciences, Central South University, Changsha, China; b Xinjiang Medical University, Urumqi, China

**Keywords:** Forensic sciences, dental age estimation, Cameriere’s method, South China, linear regression

## Abstract

The aim of this study was to evaluate the applicability of Cameriere’s European formula for age estimation in children in South China and to adapt the formula to establish a more suitable formula for these children. Moreover, the performance of dental age estimation based on Cameriere’s method combining the developmental information of permanent teeth (PT) and third molar (TM) was also analysed. Orthopantomographs of 720 healthy children in Group A, and orthopantomographs of 320 children and 280 subadults in Group B were assessed. The samples of Group A were divided into training dataset 1 and test dataset 1, and the samples of Group B were also divided into training dataset 2 and test dataset 2. A South China-specific formula was established based on the training dataset 1, and the comparison of accuracy between the Cameriere’s European formula and the South China-specific formula was conducted with the test dataset 1. Additionally, a PT regression model, a TM regression model, and a combined regression model (PT + TM) were established based on the training dataset 2, and the performance of these three models were validated on the test dataset 2. The Cameriere’s European formula underestimated chronological age with a mean difference (ME) of −0.47 ± 1.11 years in males and −0.69 ± 1.19 years in females. However, the South China-specific formula underestimated chronological age, with a mean difference (ME) of −0.02 ± 0.71 years in males and −0.14 ± 0.73 years in females. Compared with PT model and TM model, the PT and TM combined model obtained the smallest root mean square error (RMSE) of 1.29 years in males and 0.93 years in females. In conclusion, the South China-specific formula was more suitable for assessing the dental age of children in South China, and the PT and TM combined model can improve the accuracy of dental age estimation in children.

**Key points**
- Orthopantomographs of 720 healthy children in Group A, and orthopantomographs of 320 children and 280 subadults in Group B were assessed.
- A South China-specific formula was established based on the training dataset 1, and the comparison of accuracy between the Cameriere’s European formula and the South China-specific formula was conducted with the test dataset 1.
- A PT regression model, a TM regression model, and a combined regression model (PT + TM) were established based on the training dataset 2, and the performance of these three models were validated on the test dataset 2.
- The South China-specific formula was more suitable for assessing the dental age of children in South China, and the PT and TM combined model can improve the accuracy of dental age estimation in children.

## Introduction

Nowadays, chronological age (CA) estimation for children and adolescents involving the assessment of dental characteristics is widely used, as dental characteristics are more reliable and under genetic controlled than other morphologic features [1–3]. Dental characteristics include tooth mineralization, tooth eruption, abrasion, secondary dentin deposition, and root reabsorption. The radiographic evaluation of teeth mineralization and assessment of tooth eruption are the two main methods of CA estimation [4,5]. As the eruption of permanent teeth (PT) is easily affected by environmental factors, such as eating habits, nutrition status, local trauma, and persistence of deciduous teeth [3,6,7], recent studies of CA estimation based on radiographically observed that teeth mineralization has wider application in both forensic medicine and clinical oral medicine [8–10].

In the context of forensic medicine, it is common to use CA estimation to determine whether an individual is at or above the age of criminal responsibility, identify individuals in mass disasters, and estimate the age of adopted children with false birth certificates or asylum seekers of unknown age [11,12]. In clinical oral medicine, the purpose of CA estimation is to help ascertain diagnoses and plan treatments; additionally, CA estimation plays a significant role in pediatric endocrinology and orthodontic treatment [12].

In 2006, Cameriere et al. [13] proposed a new method to estimate CA in Italian children based on the relationship between age and measurement of open apices in tooth roots. Later, the authors collected and analysed samples from 2 652 Caucasian children from seven European countries, and then developed a European formula for CA estimation [14]. Several previous studies have reported the performance of Cameriere’s method for estimating CA in children in Colombia, Malaysia, Italy, and Turkey [15–19]. But the results of these studies indicate that the accuracy of dental age estimation using Cameriere’s method in different populations is varied. A regression formula was needed for this specific population, as Cameriere’s method was developed based on data from seven European countries, and it may not be optimal for other ethnic groups.

Moreover, to improve the accuracy of dental age estimation in children, some scholars have proposed a combination of developmental information on PT and third molar (TM) to estimate dental age [20–24]. The performance of the combined model has been analysed in United Arab Emirati [20], Brazilian [21], Japanese [22], Malaysian [23] and Somali children [24]. But there are no reports about the performance of the combined model in children in South China. Thevissen et al. [25] have evaluated nine methods (Gleiser, Hunt; Haavikko; Demirjian; Raungpaka; Gustafson, Koch; Harris, Nortje; Kullman; Moorrees; Cameriere) for dental age estimation based on the TM, and found that the Moorrees’ method was the most accurate due to the more detailed tooth development stages. So, the Moorrees’ method may be suitable for evaluating the TM development stage in our study.

The aim of this study was to evaluate the applicability of Cameriere’s European formula for age estimation in children in South China and to adapt the formula to establish a more suitable formula for these children. Moreover, the performance of dental age estimation based on Cameriere’s method and Moorrees’ method combining the developmental information of PT and TM was also analysed.

## Materials and methods

### Sample collection

A total of 803 orthopantomograms (OPGs) (400 males and 403 females) of healthy children (aged from 4.00 to 15.99 years) and 280 OPGs (140 males and 140 females) of healthy subadults (aged from 16.00 to 22.99 years) taken between June 2017 and November 2018 were obtained with official approval from the Xiangya Stomatological Hospital, which was affiliated with Central South University, China (Table 1). The inclusion criteria involved good-quality OPGs of healthy children and subadults who grew up in South China. The exclusion criteria involved OPGs of children and subadults with hypodontia, pathological diseases, genetic anomalies, and previous or current orthodontic treatment.

**Table 1.** Age and sex distribution of the whole studied population.

Groups (years)	Males	Females	Total
4.00–4.99	30	30	60
5.00–5.99	30	30	60
6.00–6.99	30	30	60
7.00–7.99	30	30	60
8.00–8.99	44	44	88
9.00–9.99	39	41	80
10.00–10.99	36	36	72
11.00–11.99	34	31	65
12.00–12.99	31	36	67
13.00–13.99	32	31	63
14.00–14.99	32	33	65
15.00–15.99	32	31	63
16.00–16.99	20	20	40
17.00–17.99	20	20	40
18.00–18.99	20	20	40
19.00–19.99	20	20	40
20.00–20.99	20	20	40
21.00–21.99	20	20	40
22.00–22.99	20	20	40
Total	540	543	1 083

### Information record

All OPGs were randomly numbered, and the gender, date of birth and date of OPGs were all recorded. The CA of each child at the time of the OPG was calculated based on the date of birth and date of the OPGs. The samples were randomly divided into two groups: Group A of 720 children, and Group B of 320 children and 280 subadults (Table 2). The samples of Group A all had seven PT of the left mandible and then been randomly divided into training dataset 1 (288 males and 288 females) and test dataset 1 (72 males and 72 females) according to the radio of 8:2. Similarly, the samples of Group B had seven PT of the left mandible and at least one third molar, and then been randomly divided into training dataset 2 (240 males and 240 females) and test dataset 2 (60 males and 60 females) according to the ratio of 8:2. The numbers of samples are the same in all age groups and both genders.

**Table 2.** Age and sex detailed distribution (*n*) of the training set and test set.

Groups (years)	Training set 1		Test set 1		Training set 2		Test set 2	
	Males	Females	Males	Females	Males	Females	Males	Females
4.00–4.99	24	24	6	6	0	0	0	0
5.00–5.99	24	24	6	6	0	0	0	0
6.00–6.99	24	24	6	6	0	0	0	0
7.00–7.99	24	24	6	6	0	0	0	0
8.00–8.99	24	24	6	6	16	16	4	4
9.00–9.99	24	24	6	6	16	16	4	4
10.00–10.99	24	24	6	6	16	16	4	4
11.00–11.99	24	24	6	6	16	16	4	4
12.00–12.99	24	24	6	6	16	16	4	4
13.00–13.99	24	24	6	6	16	16	4	4
14.00–14.99	24	24	6	6	16	16	4	4
15.00–15.99	24	24	6	6	16	16	4	4
16.00–16.99					16	16	4	4
17.00–17.99					16	16	4	4
18.00–18.99					16	16	4	4
19.00–19.99					16	16	4	4
20.00–20.99					16	16	4	4
21.00–21.99					16	16	4	4
22.00–22.99					16	16	4	4
Total	288	288	72	72	240	240	60	60

### OPGs measurement

OPGs were exported in .jpg format and the exported images were saved in a computer file. The images were then processed by a computer-aided image editing programme (Adobe Photoshop 7; Adobe Inc., San Jose, CA, USA). All OPGs were assessed by two different researchers and only the number of OPGs was provided. The seven PT of the left mandible were measured using the Cameriere’s method, but the TM were staged using Moorrees’ method [26]. After 4 weeks, a randomly selected sample of 100 OPGs was used to assess intra-observer reliability based on the intra-class correlation coefficient (ICC) and kappa statistic. Additionally, another sample of 100 OPGs was assessed by the first observer and another observer to assess inter-observer reliability based on the ICC and kappa statistic.

### Formula establishment

A South China-specific formula was established based on the training dataset 1. The normalized measurements (*x_i_*, *i =* 1,…,7), sum of normalized measurements (*s*), number of teeth with closed apices (*N*_0_), and gender (*g*) were the independent variables, and CA was the dependent variable. Then a ridge regression formula was conducted with R software (https://www.r-project.org) to solve the multicollinearity among variables. The ridge regression weakened the multicollinearity through partial least-square regression. Additionally, a permanent teeth regression model (PT), a third molar regression model (TM), and a permanent tooth and third molar combined regression model (PT + TM) were established based on the training dataset 2. It was noting that the development stages of TM evaluated by the Moorrees’ method was converted to ordinal categorical variable as an independent variable in the TM and PT + TM model. After collinearity diagnostics, the PT, TM and PT + TM needed to use ridge regression method based on R software.

### Accuracy comparison

The comparison of accuracy between the Cameriere’s European formula and the South China-specific formula was conducted with the test dataset 1. The mean error (ME) between the CA and the dental age (DA) was analysed to assess the direction of the error of CA estimations (overestimation or underestimation). Mean absolute error (MAE) and root mean square error (RMSE) were used to compare the accuracy between these three formulas. Paired-samples *t* test or Wilcoxon’s signed rank test was conducted to analyse the significance of differences between CA and DA for the three formulas.

Furthermore, the PT regression model, the TM regression model and the combined regression model were validated on the test dataset 2. The ME, MAE and RMSE of the three-regression model were also analysed. To assess the detailed age estimation performance, the RMSE of the three-regression model for all age groups and both sexes were presented. The statistical analysis was carried out using SPSS version 20.0 (IBM, Armonk, NY, USA) and the threshold considered statistically significant was 5%.

## Results

There were no significant intra- or interobserver differences in measurements, with ICCs of 0.992 and 0.981 for Cameriere’s method, respectively. Similarly, the value of kappa statistic of intra- and interobserver agreements were 0.980 and 0.962 for Moorrees’ method.

To improve the accuracy of CA estimation in children in South China and solve the multicollinearity among variables, a new ridge regression formula was developed using the training dataset 1. This regression formula included the following significant variables: gender (*g*; 1 for boys and 0 for girls), normalized open apex width of the canine (*x*_3_) and the first premolar (*x*_4_), number of teeth with closed apices (*N*_0_), sum of normalized measurements (*s*) and the first-order interaction between *s* and *N*_0_. The data yielded the following ridge regression formula (1) for children in South China:

(1) $$\text{DA}=\text{1}0.\text{575}+0.\text{343}g-\text{2}.\text{6}0\text{5}x_{\text{3}}-\text{2}.\text{343}x_{\text{4}}+0.\text{594}N_{0}-0.\text{416}s-0.\text{17}0s\cdot N_{0}.$$

To verify the utility of the new ridge regression formula, the Cameriere’s European formula (2) was also obtained.

(2) $$\text{DA}=\text{8}.\text{387}+0.\text{282}g-\text{1}.\text{692}x_{\text{5}}+0.\text{835}N_{0}-0.\text{116}s-0.\text{139}s\cdot N_{0}.$$

To compare the accuracy of the two formulas, the ME, MAE and RMSE for males and females were analysed in the test dataset 1 (Table 3). For males, the CA was 9.97 ± 3.40 years, and the DA was 9.50 ± 2.89 years based on the Cameriere’s European formula and 9.95 ± 3.48 years based on the ridge regression formula. So the ME of ridge regression formula for males was the smallest (−0.02 ± 0.71 years), followed by Cameriere’s European formula (−0.47 ± 1.11 years). The MAE of ridge regression formula was smaller than that of Cameriere’s European formula (Ridge: 0.52 years; European: 1.00 years). The RMSE of ridge regression formula for males was the smallest (0.69 years) but the RMSE of Cameriere’s European formula was the largest (1.17 years). For females, the CA was 10.00 ± 3.40 years. The ridge regression formula obtained smaller ME, MAE and RMSE (ME: −0.14 ± 0.73 years; MAE: 0.58 years; RMSE: 0.74 years), with the DA of 9.87 ± 3.17 years. But the DA of Cameriere’s European formula for females was 9.32 ± 2.65 years. So the ME, MAE and RMSE of Cameriere’s European formula was larger than that of ridge regression formula (European: ME: −0.69 ± 1.19 years, MAE: 1.19 years; RMSE: 1.37 years). Moreover, the larger difference in ME, MAE and RMSE (difference in ME: 0.45 years (M), 0.55 years (F) and 0.50 years (Total); difference in MAE: −0.49 years (M), −0.61 years (F) and −0.55 years (Total); difference in RMSE: −0.48 years (M), −0.63 years (F) and −0.56 years (Total)) existed between the ridge regression formula and Cameriere’s European formula, which were statistically significant (Table 4).

**Table 3.** Summary of mean differences between dental age (DA) and chronological age (CA) from Cameriere's European formula and the South Chinese formula based on ridge regression for males and females in the test dataset 1 (144 OPGs).

Gender	Number	CA (±SD)	DA (±SD)	ME (±SD)	95%CI	MAE (±SD)	RMSE	*P*-value
Males	72	9.97 (±3.40)	9.50 (±2.89)	−0.47 (±1.11)	−0.73 to −0.21	1.00 (±0.65)	1.17	0.00*
			9.95 (±3.48)	−0.02 (±0.71)	−0.19 to 0.15	0.52 (±0.48)	0.69	0.82
Females	72	10.00 (±3.40)	9.32 (±2.65)	−0.69 (±1.19)	−0.97 to −0.41	1.19 (±0.67)	1.37	0.00*
			9.87 (±3.17)	−0.14 (±0.73)	−0.31 to 0.04	0.58 (±0.45)	0.74	0.12
Total	144	9.99 (±3.39)	9.41 (±2.76)	−0.58 (±1.15)	−0.77 to −0.39	1.10 (±0.66)	1.28	0.00*
			9.91 (±3.31)	−0.08 (±0.72)	−0.20 to 0.04	0.55 (±0.47)	0.72	0.20

ME: mean error; SD: standard deviation; CI: confidence interval; MAE: mean absolute error; RMSE: root mean square error.

*P*-value: obtained using paired samples *t* test or Wilcoxon's signed rank test.

* Values that showed significant difference.

**Table 4.** Comparison of the accuracy of age estimation between the South Chinese formula based on ridge regression and Cameriere's European formula for males and females in the test dataset 1 (144 OPGs).

Gender	Number	Difference in ME (SD)	*P*-value	Difference in MAE (SD)	*P*-value	Difference in RMSE
Males	72	0.45 (0.96)	0.00*	−0.49 (0.64)	0.00*	−0.48
Females	72	0.55 (0.85)	0.00*	−0.61 (0.62)	0.00*	−0.63
Total	144	0.50 (0.90)	0.00*	−0.55 (0.63)	0.00*	−0.56

ME: mean error; MAE: mean absolute error; SD: standard deviation; RMSE: root mean square error.

*P*-value: obtained using paired samples *t* test or Wilcoxon's signed rank test.

* Values that showed significant difference.

The assessment of the age estimation accuracy between the PT model, the TM model and the combined model were shown in Table 5. For males, the ME of these three models ranged from −0.08 (PT) to 0.42 years (PT + TM). The largest MAE and RMSE for males were obtained in PT model (MAE: 1.60 years; RMSE: 2.04 years), while the combined model presented the best performance of dental age estimation (MAE: 0.80 years; RMSE: 1.29 years). For females, the ME based on PT model, TM model and combined model was 0.02, 0.37 and 0.18 years, respectively. But the combined model also had the smallest MAE and RMSE for females (MAE: 0.69 years; RMSE: 0.93 years). The largest MAE and RMSE for females were obtained in the PT model (MAE: 1.56 years; RMSE: 1.98 years). The difference in ME, MAE and RMSE between the three models was listed in the Table 6. The largest differences in MAE and RMSE exist between the PT + TM and PT model (difference in MAE: −0.80 years (M), −0.87 years (F) and −0.84 (Total), and difference in RMSE: −0.75 years (M), −1.05 years (F) and −0.89 (Total)), which were statistically significant. While the differences in MAE and RMSE between the PT + TM and TM model were smaller (difference in MAE: −0.07 years (M), −0.23 years (F) and −0.15 (Total), and difference in RMSE: −0.02 years (M), −0.24 years (F) and −0.12 (Total)). The differences in MAE and RMSE between PT and TM model were middle (difference in MAE: 0.73 years (M), 0.65 years (F) and 0.69 (Total), and difference in RMSE: 0.73 years (M), 0.81 years (F) and 0.77 (Total)).

**Table 5.** Dental age estimation performances of regression models using the permanent teeth (PT), the third molars (TM) and the PT and TM combined for males and females in the test dataset 2 (120 OPGs).

	PT	TM	PT + TM
Males			
ME	−0.08	0.39	0.42
MAE	1.60	0.87	0.80
RMSE	2.04	1.31	1.29
Females			
ME	0.02	0.37	0.18
MAE	1.56	0.92	0.69
RMSE	1.98	1.17	0.93

ME: mean error; MAE: mean absolute error; RMSE: root mean square error.

**Table 6.** Comparison of the accuracy of age estimation between different regression models for males and females in the test dataset 2 (120 OPGs).

Gender	Number	Formula	Difference in ME (SD)	*P*-value	Difference in MAE (SD)	*P*-value	Difference in RMSE
Males	60	PT + TM *vs.* PT	0.50 (1.49)	0.01*	−0.80 (1.20)	0.00*	−0.75
		PT + TM *vs.* TM	0.03 (0.59)	0.71	−0.07 (0.44)	0.22	−0.02
		PT *vs.* TM	−0.47 (1.64)	0.03*	0.73 (1.25)	0.00*	0.73
Females	60	PT + TM *vs.* PT	0.15 (1.69)	0.48	−0.87 (1.30)	0.00*	−1.05
		PT + TM *vs.* TM	−0.19 (0.69)	0.03*	−0.23 (0.63)	0.01*	−0.24
		PT *vs.* TM	−0.35 (2.00)	0.18	0.65 (1.54)	0.00*	0.81
Total	120	PT + TM *vs.* PT	0.32 (1.59)	0.03*	−0.84 (1.25)	0.00*	−0.89
		PT + TM *vs.* TM	−0.08 (0.65)	0.17	−0.15 (0.54)	0.00*	−0.12
		PT *vs.* TM	−0.41 (1.82)	0.02*	0.69 (1.40)	0.00*	0.77

PT: permanent teeth 31–37; TM: third molars; ME: mean error; MAE: mean absolute error; SD: standard deviation; RMSE: root mean square error.

*P*-value: obtained using paired samples *t* test or Wilcoxon's signed rank test.

* Values that showed significant difference.

**Figure 1.**
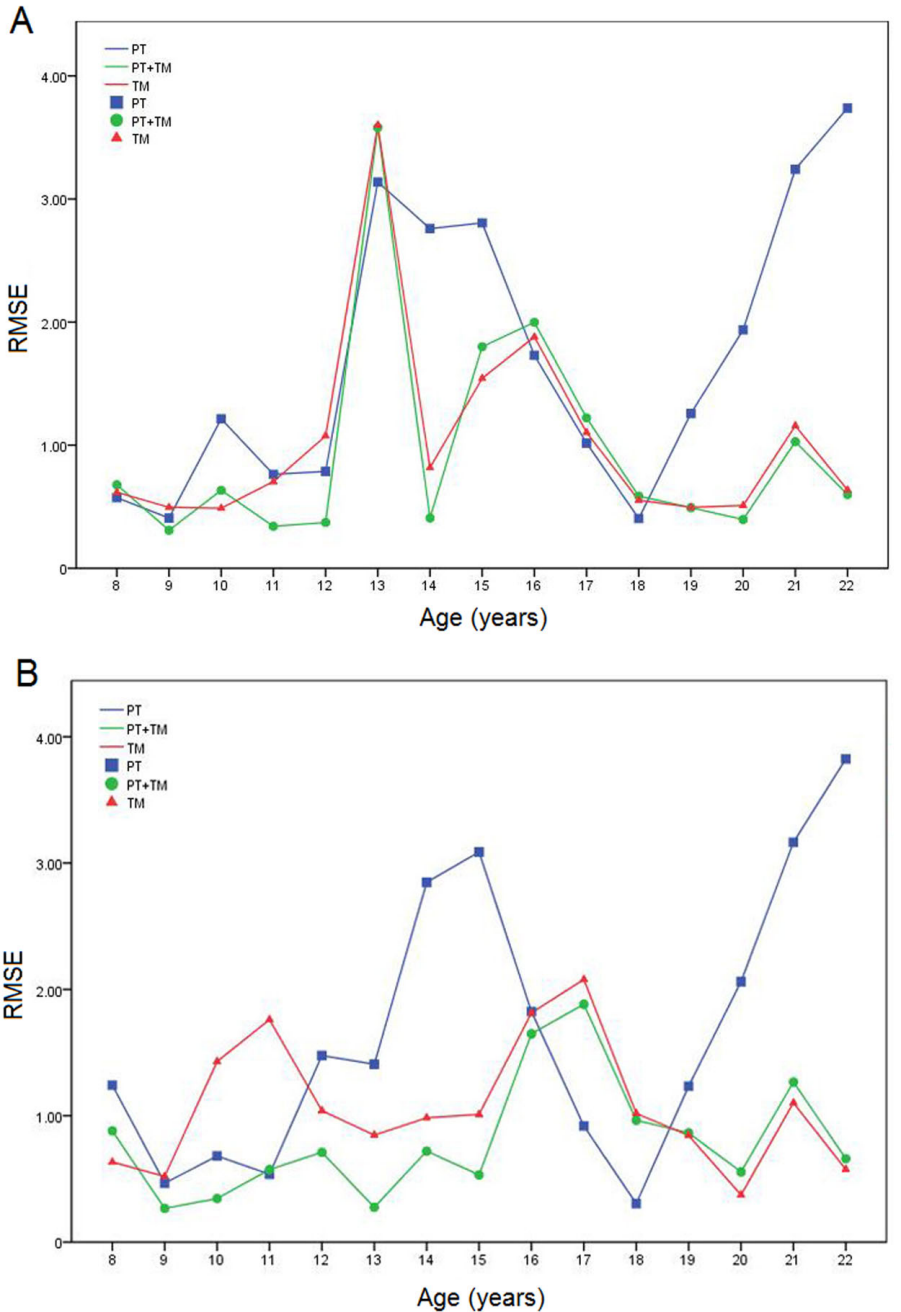
The root mean square error (RMSE) of permanent teeth (PT) model, third molars (TM) model and PT + TM model of males (A) and females (B) for all age groups in the test dataset 2 (120 OPGs).

The RMSE of these three models for all age groups and both sexes were presented in Figure 1. For PT model in males, the smaller RMSE was obtained in age group 8–12, and the RMSE of age group 18 greatly decreased. The RMSE of PT + TM and TM model was similar in males, but the RMSE of PT + TM model greatly decreased in age group 11, 12 and 14. For females, the PT and PT + TM model had smaller RMSE in younger age groups, and the smaller RMSE in older age groups was obtained by the TM and PT + TM model. A greatly decrease of RMSE could be seen in the age group 12–15 of females based on the PT + TM model. So the combined model could better help resolve criminal and civil cases involving age estimation.

## Discussion

Accurate age determination is essential in many situations, such as adoption, employment, marriage, and other situations related to social responsibilities [27]. Additionally, in the field of forensic medicine, determination of the CA of unidentified corpses is often needed [28]. Moreover, when determining the criminal responsibility of individuals who are undocumented or missing legal identification, CA determination is necessary due to individuals of different ages having different legal responsibilities according to Chinese law. Therefore, accurate CA determination is becoming increasingly important in many fields, and thus accurate and precise methods are necessary.

The Study Group of Forensic Age Diagnostics has proposed several main measures to estimate CA, comprising physical examination, dental analysis based on OPGs, X-ray examination of the left hand and wrist and conventional radiographic examination or CT scan of the clavicles after the hand ossification completed [29]. It is generally accepted that teeth development is less influenced by environmental factors compared to the development of other anatomical features [17]. Hence, CA estimation methods involving OPG-based morphological dental parameters are the most popular CA estimation methods among diverse populations [30–32]. However, when applying these methods to populations that are different from the one used to develop the method, the results tend to show significant under- or overestimation of CA [33]. Hence, it is necessary to create region-specific formulas to increase the accuracy of these methods in different regions.

Cameriere’s European formula for DA is based on the measurements of open apices of the seven left PT. As this method only requires open apices and teeth length to be measured, it is easier, and more objective compared to other quantitative methods. Some previous study showed that Cameriere’s method is more accurate than the widely used Demirjian’s or Willems’ methods [34–40].

In the present study, Cameriere’s European formula was adapted based on the training dataset 1. To weaken the multicollinearity through partial least-square regression, a ridge regression formula was established. Our results showed that the canine and first premolar were important to the model fit and were therefore included in the adapted regression formula. In a previous study in North China, in addition to the canine and first premolar, the second molar was included as a major contributor to the regression formula [41]. In a study by Halilah et al. [42] in North Germany only the canine significantly contributed to the model fit. However, unlike in the above studies, in a study conducted in Brazil, the second premolar contributed significantly to the regression formula, as in Cameriere’s European formula [43].

The comparison of accuracy of Cameriere’s European formula and South China-specific formula was examined in test dataset 1. The ME, MAE and RMSE in both genders were significantly smaller for the South China-specific formula than Cameriere’s European formula. For Cameriere’s European formula, the ME was −0.47 ± 1.11 years in males and −0.69 ± 1.19 years in females. While the ME of South China-specific formula was only −0.02 ± 0.71 years in males and −0.14 ± 0.73 years in females. The difference between Cameriere’s European formula and South China-specific formula was significant. Due to the different genetic and ethnic background, the Cameriere’s European formula based on the Italian reference dataset is not suitable for dental age estimation in children in South China. It is necessary to establish a South China-specific formula to improve the accuracy of dental age estimation in children in South China.

After combining the developmental information of PT and TM, the RMSE of PT + TM model had a decrease of 0.89 years compared with PT model and a decrease of 0.12 years compared with TM model. The PT model and TM model had larger RMSE because the PT model was suitable for children with the open apices of seven PT and the TM model was more suitable for subadult with the TM development. The combined model greatly improved the accuracy of age group 11, 12 and 14 in males and age group 12–15 in females. The reason may be that the age of subjects with at least one closed apex in our study was from 11–12 years, and most of subjects in age group 15–16 had seven closed apices in our researched population. Therefore the accuracy of dental age estimation in age group 12–15 may be improved due to the combination of developmental information of PT and TM. These results of this study are similar to the study in United Arab Emirati [20], Brazilian [21], Japanese [22] and Somali children [24]. There was an increase of RMSE in age group 13 of males for all three models because of sampling error. For one sample in age group 13 of males, the apices of seven PT were closed and the stages of TM based on the Moorrees’ method was 12, which may affect the accuracy of dental age estimation for these three models. After deleting this sample, the RMSE in age group 13 of males was the smallest using PT + TM model (PT + TM: 1.26), followed by TM model and PT model (TM: 1.52; PT: 1.97). As for age group 15 of males, the most subjects from our study have the closed apices of seven PT, so the TM model obtain the smallest RMSE. Moreover, the greatly decrease in age group 18 of both genders may be due to the point estimation which means the dental age for subjects with closed apices of seven PT was 18.43 years using PT model. The accuracy of dental age estimation in children in south China can be improved by combining the developmental information of PT and TM, which could better help resolve criminal and civil cases involving age estimation.

There are no previous studies adapting Cameriere’s European formula to children in South China. However, in 2015, Guo et al. [41] adapted Cameriere’s European formula for samples from North China and the influential variables in their formula were different from those in the present study. When the formula from North China was applied in our studied population, the ME, MAE and RMSE were greatly larger than that obtained by our South China-specific formula (North China: ME: −1.04 years, MAE: 1.14 years, RMSE: 1.40 years; South China: ME: −0.08 years, MAE: 0.55 years, RMSE: 0.72 years). This discrepancy may be due to the fact that dental development is influenced by genetic, socioeconomic, and environmental factors [44]. A study of dental age estimation in western Turkish children suggested that the degree of dental maturity in western Turkish children was lower than that in the eastern, northeastern and northern Turkish children [45]. Baylis and Bassed [46] also indicated that the differences of dental developmental rates were observed between the different regions within the New Zealand. In a country as large and diverse as China, there is no doubt that different districts have different genetics, ethnic backgrounds, socioeconomic status, dietary habits, and nutrition. Therefore, even in the same country, the dental development of different populations varies. Further studies using larger sample sizes and younger age groups are recommended to evaluate the applicability of the South China-specific formula in other specific populations and to compare the new formula with other well-established CA estimation methods.

## Conclusion

Compared with the Cameriere’s European formula, the South China-specific formula was more suitable for estimating the dental age of children in South China. Compared with PT model and TM model, the PT and TM combined model can improve the accuracy of dental age estimation in children of 12–15 years.
